# The Application of Wearable Technology to Quantify Health and Wellbeing Co-benefits From Urban Wetlands

**DOI:** 10.3389/fpsyg.2019.01840

**Published:** 2019-08-13

**Authors:** Jonathan P. Reeves, Andrew T. Knight, Emily A. Strong, Victor Heng, Chris Neale, Ruth Cromie, Ans Vercammen

**Affiliations:** ^1^Wildfowl & Wetlands Trust (WWT), Slimbridge, United Kingdom; ^2^Department of Life Sciences, Imperial College London, Ascot, United Kingdom; ^3^Department of Botany, Nelson Mandela University, Port Elizabeth, South Africa; ^4^The Silwood Group, London, United Kingdom; ^5^Frank Batten School of Leadership and Public Policy, University of Virginia, Charlottesville, VA, United States; ^6^Centre for Environmental Policy, Imperial College London, London, United Kingdom

**Keywords:** blue–green space, wellbeing, stress, salutogenesis, physiology, electroencephalogram (EEG), psychological restoration, blue–green infrastructure

## Abstract

Improved nature provision in urban environments offers great potential for achieving both biodiversity conservation and public health objectives. Yet there are few experimental studies that address links between specific natural environments and physiological and/or psychological changes that could contribute to the health and wellbeing co-benefits of urban nature. In addition, relative to green space, the salutogenic impact of aquatic environments are understudied. Here, we present a feasibility study examining the use of low-cost wearable technology to quantify the psychophysiological effects of short-term exposure to urban wetlands. The study took place at the WWT London Wetland Centre, which is characterized by its contrasting biodiverse wetland habitat and surrounding urban setting. Thirty-six healthy participants experienced counterbalanced exposures to an indoor space, a wetland, an urban site. We continuously recorded electroencephalographic (EEG) data and real-time physiological stress responses; with additional monitoring of post-exposure self-reported mood states. We found a significant effect of site on mean resting heart rate (HR), with increased HR in the urban setting, although this was only observed in participants with pre-existing high stress. We found no significant differences in other measures of physiological stress responses (heart rate variability and electrodermal activity). The EEG data showed modulation of high beta band activity only in the wetland setting, potentially related to changes in attention. However, the EEG findings were confounded by low quality signals and artifacts caused by movement and environmental interference. Assessments of self-reported mood states demonstrated an increase in positive feelings in the wetland setting. A pronounced decrease in negative feelings in the wetland setting was observed in stressed individuals only. Our results suggest that pre-existing stress levels may be an important modulator of the salutogenic effect of blue-green space. We provide partial support for the hypothesis that exposure to blue-green space promotes stress recovery and for the use of low-cost psychophysiological measurements to quantify the potential stress-reducing effects of blue–green space exposure in urban dwellers. Further technological refinement is required for this approach to become a viable tool to support evidence-based decision-making for public health and green/blue space provision.

## Introduction

It is estimated that by 2050 two-thirds of the global human population will live in urban centers (United Nations Department of Economic and Social Affairs, 2014). Future urban environments are predicted to face significant challenges associated with increased air and water pollution, altered water regimes, diminished water quality, greater extremes of flood and drought (Taylor et al., 2004; Miller and Hutchins, 2017) and urban heat island effects (Levermore et al., 2018). Therefore the trend toward urbanization, especially in the context of climate change, poses significant challenges to the many activities that influence people’s health and wellbeing (HWB), including urban planning, infrastructure development, employment, food security and healthcare. Widespread adoption of blue-green infrastructure (BGI) within growing and emerging urban environments has the potential to secure and create ecosystem services that counter these adverse biophysical impacts of urbanization (Voskamp and Van de Ven, 2015). BGI may also provide socio-cultural and public health co-benefits through their association with increased social inclusion, interaction, and cohesion (e.g., Cohen et al., 2008; Francis et al., 2012; Hartig et al., 2014; Jennings et al., 2016; Jennings and Bamkole, 2019), improved public safety (e.g., Branas et al., 2011) and enhanced quality of life (e.g., Thompson et al., 2014; Aspinall et al., 2015; McCracken et al., 2016; Mensah et al., 2016).

In recent years, numerous studies have reported on the role of exposure to natural environments in both disease prevention and salutogenesis, the latter referring to the promotion of HWB rather than simply the absence of disease (for a recent review, see Kondo et al., 2018a). The literature suggests several possible pathways by which exposure to natural environments may deliver HWB benefits (Hartig et al., 2014; Kuo, 2015). Two prominent and complementary frameworks are the Stress Recovery Theory (SRT; Ulrich, 1983) and the Attention Restoration Theory (ART; Kaplan, 1995), both of which posit that natural environments have restorative potential, because they moderate physiological arousal or reduce mental fatigue (Berto, 2014). It is hypothesized that this reduction in psychological and physiological stress promotes HWB benefits.

Different types and qualities of natural environments influence HWB in different ways and extents (Hartig et al., 2014; South et al., 2015; Wheeler et al., 2015). Research into aquatic environments or ‘blues spaces,’ defined broadly as any environment predominantly comprising a natural or human-modified water feature, has primarily focused on coastal environments (e.g., Miller et al., 2012; Wheeler et al., 2012; White et al., 2013). Despite being prominent environmental features, freshwater blue spaces, such as lakes, ponds, rivers, streams and other wetlands, are largely understudied (White et al., 2010; Foley and Kistemann, 2015), particularly in urban areas. Despite insufficient research and substantial heterogeneity across studies, systematic reviews of the literature point to an overall positive association between exposure to blue space and improved mental health, wellbeing and the promotion of physical activity has been identified (Gascon et al., 2015, 2017). Research in Germany and Hong Kong supports the delivery of salutogenic effects by blue space provision in large cities (Völker et al., 2018; Garrett et al., 2019). In the United Kingdom, a region-wide, cross-sectional survey suggests visits to freshwater blue space promotes social interaction and conveys psychological benefits (de Bell et al., 2017). Recently announced research programs in Europe will further aim to map and quantify the impacts of urban blue infrastructure (Grellier et al., 2017). A few experimental studies have attempted to isolate the effects of blue space as compared to green space by exposing participants to photographs as a proxy for natural environments. These findings do suggest that blue space has enhanced capacity to promote psychological restoration relative to green space (e.g., White et al., 2010; Seresinhe et al., 2015; Grassini et al., 2019).

Historically, some sectors of the nature conservation movement have promoted approaches to preserving natural environments by excluding people from them or restricting access through the establishment of protected areas (Brockington and Igoe, 2006). This conservation method stands in stark contrast to the more recent narrative that favors (re)connecting people with high quality and/or extensive natural environments to foster resilience in both ecosystems and human communities (e.g., Wood et al., 2017). Research in the health sciences and increasingly from conservation biology, environmental psychology, environmental education and sustainability science further supports the beneficial effects of (re)connecting people with nature to enhance human HWB and promote the uptake of pro-environmental attitudes and behaviors (Ives et al., 2018; Rosa et al., 2018). Despite a growing interest in understanding how exposure to natural environments might be leveraged to achieve the goals of both conservation and public health (Bottrill et al., 2014; Milner-Gulland et al., 2014; Lovell et al., 2015), progress in this field has been limited by the complexities of human–environment interactions and a lack of understanding of the mechanisms that support the delivery of benefits (Hartig et al., 2014; Shanahan et al., 2015; Dadvand et al., 2016). Retrospective and observational studies dominate the literature and few studies have focused specifically on inland aquatic environments. Laboratory-based, but in particular ‘natural’ experimental studies have been proposed as an important step toward developing a deeper understanding of how natural environments might be leveraged to deliver multiple co-benefits (Frumkin et al., 2017; Gascon et al., 2017). To this end, innovative methods are required to accurately capture physiological and psychological responses *in situ*, as the experience unfolds (Frumkin et al., 2017). In recent years, we have seen a significant rise in the popularity and quality of a wide range of commercially available wearable technologies that allow for continuous monitoring of physiological signals (Bunn et al., 2018). As they are minimally obtrusive and offer opportunity to observe individuals in ‘naturalistic’ outdoor experiments, these devices are increasingly used in research applications. A small number of studies have used mobile devices to monitor responses to urban versus natural environments (Kondo et al., 2018b but see: Aspinall et al., 2015; Neale et al., 2017; Tilley et al., 2017; Olszewska-Guizzo et al., 2018) but – to our knowledge – none have specifically focused on blue space.

**FIGURE 1:**
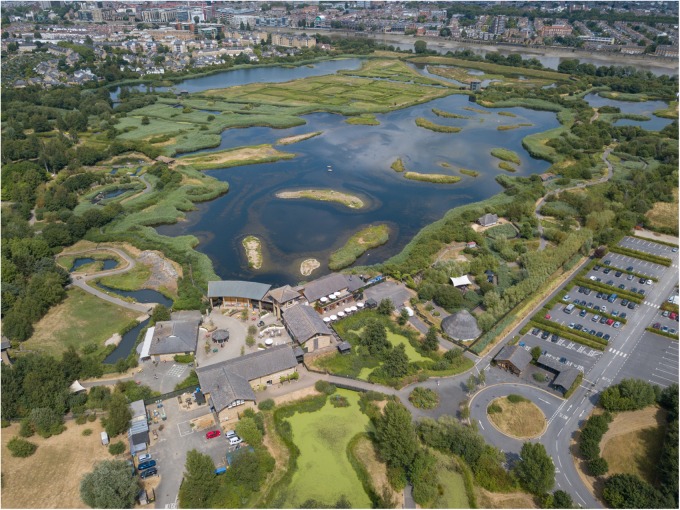
An image of the study site, featuring the WWT London Wetland Centre and the urbanized area of West London (Barnes). Photograph credits: Sam Stafford (WWT).

In this study we take advantage of the unique setting of the WWT London Wetland Centre, a 42 hectare constructed urban wetland in West London, created and managed by the Wildfowl & Wetlands Trust (WWT)^1^ to experimentally test, for the first time, the salutogenic potential of urban wetlands. As a wetland conservation charity, WWT seeks to provide co-benefits to biodiversity and to the broader public through recreation and education activities at its wetland conservation sites. WWT London Wetland Centre visitor feedback frequently describes the site as an ‘oasis’ within the metropolis of London (Figure 1). To explore this further, WWT engaged in a collaboration with Imperial College London to investigate and develop metrics for monitoring and evaluating the potential HWB benefits delivered to visitors. Specifically, we aimed to critically evaluate the feasibility of low-cost wearable technology to complement subjective measures of HWB. We report on a pilot study implementing a commercially available EEG system and photoplethysmography (PPG) device to detect differences in brain activity and physiological stress as a short-term wetland visitor experience unfolded. This represents an important advance upon previous research in this field, which is typically limited to broad-scale epidemiological or laboratory-based experiments using only proxies for real blue space exposure. The findings are intended to inform the design, monitoring, evaluation and improvement of WWT activities aimed at delivering HWB benefits to communities and visitors, and, more generally, to support evidence-based policy-making in urban planning (Wildfowl & Wetlands Trust [WWT], 2017).

## Materials and Methods

### Participants

We recruited 36 participants for this study, the majority of which were contacted through a social corporate responsibility scheme which allows local professionals to volunteer for WWT during normal work hours. Just over half (53%, *n* = 34) had never visited the wetland center before. Of the participants who gave responses, 83% (*n* = 29) were not WWT members or affiliates, while 68% percent (*n* = 25) were not members of other conservation charities. Overall, the majority of our sample was unfamiliar with wetland conservation prior to participating in the study. All participants were screened against exclusion criteria, i.e., the presence of neurological conditions or injuries, psychiatric disorders, cardiovascular disease, taking of prescription or over-the-counter medicines, and any departure from normal or corrected-to-normal eyesight or hearing. This resulted in exclusion of *n* = 2 participants. Written prior and informed consent was obtained from each participant.

### Study Site

The study took place in and around the WWT London Wetland Centre located in Barnes, West London, which was created through the conversion of four disused Victorian reservoirs into a range of biodiverse wetland habitats. The WWT London Wetland Centre consists of a visitor center (including a café and education center), an area of living collection of wetland birds and mammals, and surrounding managed wetland habitats. The latter carries the Site of Special Scientific Interest (SSSI) designation due to its national importance for UK breeding birds in lowland open waters and for its assemblages of non-breeding gadwall (*Anas strepera*) and shoveler (*Anas clypeata*). This site was selected for this experiment because of its unique location (Figure 1) that enables a quick transition between exposure to a species-rich SSSI wetland and a typical urban setting. The sites were selected with the intention of achieving maximal contrast between the urban and wetland environment in order to maximize the potential of detecting differences in psychophysiological and subjective responses from short exposures. Limitations around experimental practicalities were also taken into account i.e., the need to reduce distance walked and time in the experiment.

#### Control Site

**FIGURE 2:**
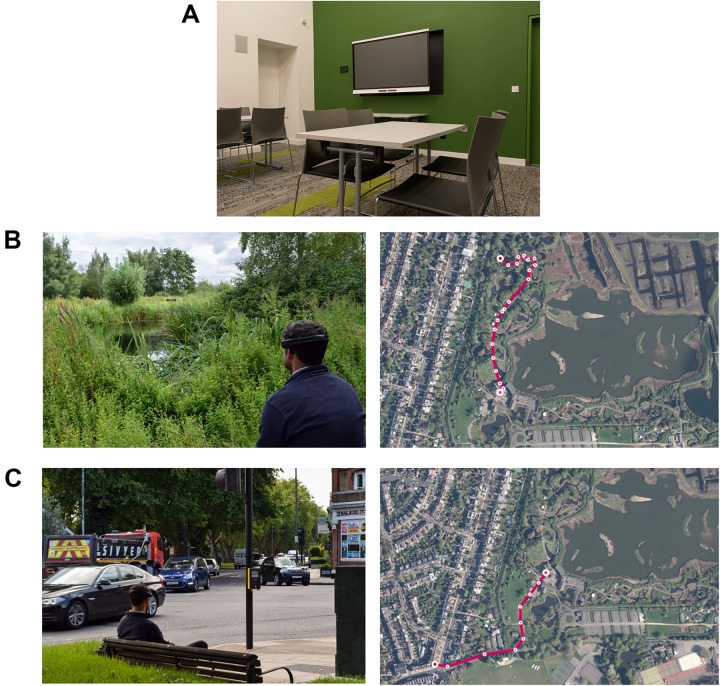
Composite image illustrating the exposure conditions, i.e., the control room where baseline measures were taken **(A)**, and representative images of the wetland setting **(B)** and the urban setting **(C)**, and aerial views of the walk to each site from the control room. Photograph credits: Sam Stafford and Kate Smith (WWT).

A multipurpose function room at WWT London Wetland Centre (51°28′37.9′′N 0°14′09.2′′W) was used as a control setting for baseline measures of PPG and EEG (Figure 2A). The room contents consisted of chairs, tables, a TV and modest décor. Its use as a function/class room enabled us to isolate participants from center visitors and external noise, thus creating a relatively low stimuli environment. It is favorably located between the wetland and urban sites.

#### Wetland Site

The control site is situated in the visitor center area (51°28′47.2′′N 0°14′08.5′′W), which includes the café, shop and education center of the WWT London Wetland Centre site. Adjacent is a captive animal area and beyond the captive area is ‘Wildside’: a managed ‘natural’ wetland area (Figure 2B). We were cognizant of the need to keep distance walked to minimum because of headset discomfort and experimental fatigue and also because our interest was in seated/resting responses to urban and natural wetland environments. For the latter reason, both the wetland and urban sites were chosen because they featured permanent benches and represented ‘real-world’ resting spots. The wetland site was chosen because it was the first bench within ‘Wildside’ with a blue/green vista overlooking water.

#### Urban Site

The study area (WWT London Wetland Centre) was chosen to create maximally opposing sensory experiences and to test differential responses to these environments. With similar consideration to the wetland site, the urban site (51°28′31.3′′N 0°14′21.3′′W) was chosen because it was the first suitable urban bench outside the Centre. Suitability was judged by the number of features present at this site that are typical of busy urban settings e.g., a traffic intersection, traffic lights, a pedestrian crossing, passers-by, shops, and cafes.

### Procedure

The study was conducted between the 14th July and 23rd August 2017. Prevailing weather conditions were recorded for each participant session (cloud cover, air temperature, wind speed humidity using UK Met Office data^2^). The level of other potentially confounding variables (e.g., air traffic) was also recorded. Over the 6 week testing period no sessions were canceled due to adverse weather. Participants attended their allocated session individually, with scheduled start times at 9am, 12pm, or 2pm. Upon arrival participants were greeted by a researcher and shown to the visitor center. We first conducted brief, semi-structured interviews to explore participants’ motivations to take part, expectations about the experiment, anticipated benefits, pre-existing knowledge of the site, any formal or informal affiliation to WWT (e.g., member, donor, volunteer), support for other conservation charities, and their understanding of the aims and procedures. Participants were also asked how they felt in the moment, and whether that was a departure from their regular mental and/or physical state. Following the interview, participants were asked to complete a set of questionnaires to obtain demographic information (age, gender, employment status), and an assessment of relevant psychological variables including life stress, recent levels of depression, anxiety and stress, and participants’ sense of connectedness with nature to provide an assessment of participants recent exposure and response to stressors. The in-depth interview data were intended to provide qualitative contextual information, but a report on this is beyond the scope of the current study.

The experimental setup consisted of a wristband measuring real-time physiological data and a mobile EEG device [for details, see Depression, Anxiety Stress Scale (DASS-21)]. Fitting the measurement equipment took approximately 15 min. Following calibration, EEG and physiological data were continuously recorded for the full duration of the experiment. Participants remained seated in the indoor control room for 10 min to obtain stable baseline readings (Figure 2A). For the outdoor sections of the experiment, participants carried a light rucksack with the EEG data acquisition laptop. A researcher accompanied the participant to each site. Walking distances between the control and the urban and wetland setting were 390 and 430 m, respectively (Figures 2B,C), the terrain was flat. Participants were then asked to sit for 10 min on a permanent bench at each site, with the researcher retiring out of sight for all exposure periods. For all three exposures, and for the full 10 min, participants were instructed to relax, remove the backpack, remain seated and to take in their surroundings, but not to close their eyes. At the end of the 10 min exposure, participants were asked to complete a self-report survey assessing mood states. After completing the baseline, urban and wetland site exposures, a short debriefing session followed. We were specifically interested in whether participants showed a stated preference for the urban or the wetland site and whether they had experienced any physical (e.g., from wearing the equipment) or psychological discomfort or distress that might confound the outcome measures.

The study had a repeated measures design, with three exposure conditions/sites: indoor (control), urban and wetland. Condition order of the urban and wetland sites was counterbalanced between participants; the control condition was always administered first.

### Baseline Assessments

#### The Holmes and Rahe Stress Inventory

The Holmes & Rahe Stress Inventory, also known as the Social Readjustment Rating Scale (Holmes and Rahe, 1967) comprises a checklist of significant life events. Respondents are asked to indicate the number of events they have experienced in the last year. Frequencies of occurrence are multiplied by the event’s ‘Life Change Units’ as an indicator of severity. The total summed scores is indicative of overall stress and associated with the risk of developing stress-related health problems.

#### Depression, Anxiety Stress Scale (DASS-21)

We used the 21-item version of the Depression, Anxiety and Stress Scale (DASS-21; Henry and Crawford, 2005) to measure reported signs of depression (expressed as dysphoria, hopelessness, devaluation of life, self-deprecation, lack of interest/involvement, anhedonia, or inertia), anxiety (expressed as autonomic arousal, muscle tension, situational anxiety, and subjective experience of anxious affect) and stress (expressed as difficulty relaxing, nervous arousal, and being easily agitated, irritable, over-reactive and impatient). Respondents are asked to rate each of the 21 items (e.g., “I found it hard to wind down”) on a 4-point Likert scale, indicating the extent to which they have experienced that state over the past week. Scores for depression, anxiety and stress are calculated by summing the scores for the relevant items. The DASS-21 has high internal consistency and yields meaningful discriminations in a variety of settings. We used the UK normative data published by Henry and Crawford (2005) to categorize participants as having either above or below average stress levels.

#### The Nature Relatedness Scale

The Nature Relatedness Scale (Nisbet et al., 2009) is a 21-item instrument used in research on sustainability and wellbeing, serving as a measure of individual differences in connectedness with nature. Comprising of three scales, the NR-self (8 items), the NR-perspective (7 items) and the NR-experience (6 items), it provides a measure of the respondent’s internalized identification with nature (i.e., feelings of personal connectedness and meaning), their external nature-related world view (i.e., a sense of agency concerning our impact on the environment) and their physical familiarity with the natural world (i.e., a desire to interact and actively engage with the natural world). High scores are associated with increased concern for the environment.

### Outcome Assessments

#### Real-Time Physiological Stress Measures

Cardiovascular and electrodermal activity metrics are commonly used to assess and monitor the relative activity of the sympathetic and parasympathetic branches of the autonomic nervous system, which are respectively associated with states of threat (fight or flight response) and calm (rest and digest). These metrics can therefore be interpreted as indicators of physiological and psychological stress (Camm et al., 1996; Jacobs et al., 2012; Kondo et al., 2018b). We used an Empatica E4 wristband (Garbarino et al., 2014) to obtain continuous physiological responses from the participants. The wristband includes a photoplethysmography (PPG) sensor to measure blood volume pulse (BVP) which is used to derive heart rate (HR) and heart rate variability (HRV), and an electrodermal activity (EDA) sensor to measure changes in skin conductance. The wristband was attached to the non-dominant hand of the participant, with PPG and EDA data recorded at 64 and 4 Hz respectively.

##### Heart rate

Heart rate increases as a result of both physical exertion and psychological stress. Evidence suggests that HR responds to acute environmental stimuli (Kjellgren and Buhrkall, 2010; Park et al., 2010; Song et al., 2013). From the continuous HR recording, we calculated the mean HR in each setting (control, wetland and urban), expressed in beats per minute (bpm). The analysis window was restricted to the central 8 min of the exposure period. Standard HR recovery testing measures HR from *peak* exercise to 2 min post-exercise (Cole et al., 1999; Shetler et al., 2001). For moderate exercise (walking), we eliminated the first and last minute to minimize the impact of cardiovascular recovery from the walk to the site, and the effects of increased movement observed in participants toward the end of the exposure period.

##### Heart rate variability

A healthy heart is not a metronome (Shaffer et al., 2014) and HRV represents the fluctuation in the time intervals between adjacent heartbeats. Typically, higher HRV values are associated with improved physiological health and self-regulatory capacity or resilience (Carney et al., 2005; Thayer et al., 2010). HRV may also be associated with psychological processes linked to prefrontal brain function, such as attention and emotion (McCraty and Shaffer, 2015). We used Kubios HRV Standard 3.1.0 (Tarvainen et al., 2008) to process the PPG data and derive HRV parameters, applying threshold-based correction to remove artifacts and ectopic beats by comparing inter-beat interval values (the time period between successive heartbeats; IBI) against a local average interval. We then used an advanced de-trending method based on smoothness priors regularization (Tarvainen et al., 2002). Applying a time-varying high-pass filter the data are smoothed to remove IBI time series non-stationarities. Samples with artifact correction >15% were discarded to minimize the impact of artifacts on analysis results (Tarvainen et al., 2008). Consequently, only 12 participants with good quality data for all three conditions were retained (Supplementary Table 1). Following the convention for short-term recording (Camm et al., 1996), we selected 5 consecutive minutes of signal 3 min into each measurement period, thereby selecting a central portion for each period. This selection process was repeated for each participant and each condition.

Typically, HRV is analyzed as a time-domain and/or frequency-domain signal. The former quantifies the variability in measurements of the IBI. Computationally more straightforward, it is more consistently applied in the existing literature (Michael et al., 2017) compared with the frequency-domain measurements that estimate the distribution of absolute or relative power into various frequency bands.

We applied the following time-domain metrics suitable for characterization of HRV based on short (i.e., 5 min) epochs: the root mean square of successive differences of the inter-beat intervals (RMSSD), the number of adjacent NN intervals that differ from each other by more than 50 ms (NN50) and the Triangular Interpolation of the NN Interval Histogram (TINN). Note that NN intervals refer to the intervals between ‘normal’ peaks, i.e., excluding abnormal beats due to measurement artifacts. For the frequency-domain, we employed the most common method to calculate HRV spectra, i.e., a Fourier transform, to derive the primary components: the low frequency (LF, 0.04–0.15 Hz) and high frequency (HF, 0.15–0.40 Hz) spectra. For further analysis, we opted to transform the data using the natural logarithm to yield an approximately normal distribution (Michael et al., 2017).

##### Electrodermal activity

The Empatica E4 sampled EDA at 4 Hz, and skin conductance (SC) was measured in micro-Siemens (μS). Due to variation in the length of EDA readings across participants, analysis windows were standardized by removing the first minute of data. The subsequent 8 min were used in the analysis.

Continuous Decomposition Analysis (CDA) was performed on EDA data in Ledalab (Benedek and Kaernbach, 2010) to decompose SC data into tonic (skin conductance level; SCL) and phasic (skin conductance response; SCR) components (Dawson et al., 2007). Event markers were imported from Empatica E4 wristbands to determine the central 8 min SC analysis window for each exposure session. For the purposes of this study, two measures representing tonic and phasic activity were used; the mean tonic activity within a treatment window, and the number of significant SCR events, respectively.

#### Electro-Encephalography (EEG)

We used an Emotiv^®^ EPOC+ EEG system to obtain brain electrical activity measurement non-invasively through the scalp. The EPOC+ has 14 channels in the international 10–20 position system (AF3, AF4, F3, F4, F7, F8, FC5, FC6, T7, T8, P7, P8, O1, and O2), with P3 and P4 as reference electrodes. To prevent electrode dehydration we added 5% glycerol to the saline solution before wetting the electrodes. Electrodes were checked for contact quality, signals were sampled at 128 Hz per channel and sent via Bluetooth to a portable laptop computer. EEG data was pre-processed using EEGLAB (Delorme and Makeig, 2004). Data from each participant were imported and analyzed individually. In EEGLAB the 14 data, and 1 marker (events), channels were defined and mapped according to the international 10–20 position system (see above). The continuous EEG signal was filtered with high and low band-pass frequencies of 0.16 and 42 hz, and then re-referenced to the average reference in EEGLAB. Using event markers the signal was segmented into control, urban and wetland epochs with extreme artifacts and poor data regions rejected through visual inspection and ICA. A Fast Fourier Transform (FFT) was applied, normalized by the number of data points of the processed recording, to extract component frequencies for all 14 sensor location on the headset. Given that the Emotiv^®^ has only 14 channels and the data sample rate is only 128 Hz, we calculated a global average for each frequency band (e.g., see: McMahan et al., 2015). Root mean square (RMS) values were obtained for each segment of data corresponding to the exposure conditions/sites (control, wetland and urban). Using an in-house MATLAB script, we extracted RMS for delta (0.5–4 Hz), theta (4–8 Hz), alpha (9–13 Hz), low beta (13–19 Hz), high beta (21–27 Hz), frequencies, each associated with different mental states and levels of consciousness (Table 1). For the analysis of the EEG data, we therefore obtained one mean value per frequency band for each individual and each site. To standardize the data before statistical analysis, we employed a min-max scaling procedure. Following inspection of the boxplot for the different EEG frequency bands, we eliminated 30 data points as outliers (Supplementary Figure 1).

**TABLE 1:** Mental states associated with different EEG frequencies (based on: Nunez PL, Srinivasan R. Electric fields of the brain: The neuro-physics of EEG, 2nd Edition. Oxford University Press, Inc., New York, NY, United States, 2006).

**Frequency**	**Associated with…**
Delta (0.5 – 4 Hz)	• Adult slow-wave sleep • States of altered consciousness
Theta (4 – 8 Hz)	• Drowsiness or idling • Deep meditation and daydreaming • Memory encoding
Alpha (9 – 13 Hz)	• Relaxation, reflection, resting state • Creative and artistic processes
Low beta (13 – 19 Hz)	• Concentration, alertness • Task-related activity
High beta (21 – 27 Hz)	• Increases in directed attention • Anxious thought, excitement

#### Self-Reported Mood States

To capture mood state changes following exposure to the different environments, participants were asked to complete the Positive and Negative Affect Schedule (PANAS; Watson et al., 1988) following each 10 min exposure session. The PANAS consists of two 10-item scales measuring positive and negative mood states. Items are rated on a 5-point scale from “not at all” to “very much.” Summed scale scores reflect independent dispositional dimensions. A high score on the negative affect scale indicates subjective distress and un-pleasurable engagement, and a low score indicating the absence of these feelings. High scores on the positive scale are associated with enthusiasm and alertness, indicating a pleasurable engagement with the environment whereas low scores are associated with lethargy and sadness. The PANAS has been shown to produce a reliable and valid measure of positive and negative mood states in a non-clinical British sample (Henry and Crawford, 2005).

### Statistical Analyses

We report descriptive statistics for the sample’s demographic characteristics and baseline assessments of stress levels and nature-relatedness. To explore the influence of demographic differences on baseline indicators of stress and nature-relatedness, we conducted t-tests (assessing gender differences) and examined Pearson correlations (assessing associations with age). We created separate general linear models for each of the continuous outcome measures, with Site (control, urban and wetland) as the within-subjects factor, while controlling for Site Order, which was entered as a between-subjects factor. To account for individual differences that could impact physiological responses, we also controlled for current (self-reported) levels of stress. For this purpose, we classified participants into ‘self-reported low stress’ and ‘self-reported high-stress’ groups on the basis of their DASS-21 stress score relative to published UK population mean scores (Henry and Crawford, 2005), and included this as a between-subjects factor. For the EEG analysis, we included frequency band (alpha, high-beta, low-beta, theta, and delta) as an additional within-subjects factor.

## Results

### Demographic Data and Baseline Assessments

Participants were on average 41 years of age (*SD* = 10.28). Women were slightly over-represented in the sample (61.8%) compared with men (38.2%). Descriptive statistics for the baseline assessments are summarized in Table 2. On average, participants’ life stress scores were relatively elevated, which is associated with increased risk of developing stress-related health conditions. A minority (15.2%) of the sample fell into the ‘low risk’ category (scores < 150), while almost half (48.5%) of the sample was classified as being at moderate risk (scores 150–300), and a significant proportion (36.4%) were at high risk (scores > 300).

**TABLE 2:** Descriptive data for the baseline psychological measurements.

			**Scale scores**			
			
		***N***	**Sample minimum**	**Sample maximum**	**Mean**	***SD***
Holmes-Rahe social readjustment rating scale		33	39	1032	287.18	204.19
DASS-21	Depression subscale	33	0	15	2.48	3.15
	Anxiety subscale	33	0	8	1.39	1.78
	Stress subscale	33	0	18	5.30	4.30
Nature relatedness scale	Self	34	21	39	31.26	4.74
	Perspective	34	19	34	28.76	4.00
	Experience	34	10	30	23.06	4.34

The DASS-21 is indicative of self-reported stress, anxiety and depression in the last week. In relation to relevant UK-based norm scores, the sample scores (on average) fell within the 58–68th percentile for Depression, the 54–69th percentile for Anxiety and the 60–69th percentile for Stress (Henry and Crawford, 2005). Participants also reported high levels of nature relatedness, with average scores similar to those who report ‘seeing themselves as an environmentalist’ (Nisbet et al., 2009). Additional analyses with respect to demographic (age, gender) differences in these baseline measures are reported in Supplementary Tables 2, 3 and Supplementary Figure 2.

### Exposure Effects on Psychophysiological Measures

#### Heart Rate

There was a significant effect of Site on mean HR, *F*(2,56) = 3.703, *p* = 0.031, η^2^ = 0.117, and a significant interaction between Site and Stress Level, *F*(2,56) = 4.848, *p* = 0.011, η^2^ = 0.148, but no interaction effect between Site and Site Order, *F*(2,56) = 0.261, *p* = 0.771, η^2^ = 0.009. Neither the main effect of Site Order, nor Stress Level was significant, *F*(1,28) = 0.846, *p* = 0.366, η^2^ = 0.029 and *F*(1,28) = 0.215, *p* = 0.647, η^2^ = 0.008, respectively.

**FIGURE 3:**
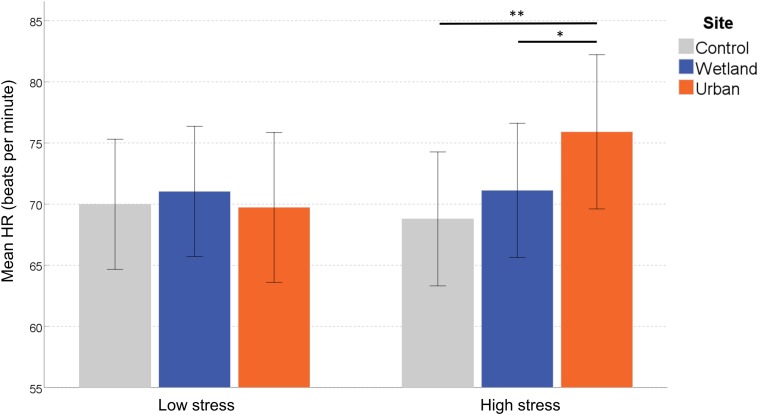
Mean heart rate (HR) at each site. Error bars indicate 95% confidence intervals. Statistical significance is indicated by ^*^p < 0.05 level, and ^∗∗^*p* < 0.01, corrected for multiple comparisons (Sidak).

To clarify the interaction effect, we conducted a simple effects analysis, which demonstrated no significant differences between sites for ‘self-reported low stress’ individuals, *F*(2,27) = 0.399, *p* = 0.675, η^2^ = 0.029, but a significant effect of Site in ‘self-reported high stress’ individuals, *F*(2,27) = 6.045, *p* = 0.007, η^2^ = 0.309. Pairwise comparisons indicated increased HR in the urban setting compared with both the wetland (*p* = 0.030) and the control setting (*p* = 0.005; corrected for multiple comparisons) (Figure 3).

#### Heart Rate Variability

We found no significant differences between the exposure conditions on any of the time-domain HRV metrics. There was no significant main effect of Site on RMSSD, *F*(2,22) = 0.711, *p* = 0.502, η^2^ = 0.061, and no effect of Site Order, *F*(1,11) = 0.29, *p* = 0.867, η^2^ = 0.003, or Stress Level, *F*(1,11) = 0.769, *p* = 0.399, η^2^ = 0.065. We observed no significant interaction effects between Site and Site Order, *F*(2,22) = 1.285, *p* = 0.297, η^2^ = 0.105, or Site and Stress Level, and *F*(2,22) = 1.781, *p* = 0.192, η^2^ = 0.139.

Similarly, the main effect of Site on NN50 was not significant, *F*(2,22) = 0.775, *p* = 0.481, η^2^ = 0.064. There was no significant main effect of either Site Order, *F*(1,11) = 0.576, *p* = 0.464, η^2^ = 0.050, or Stress Level, *F*(1,11) = 1.317, *p* = 0.275, η^2^ = 0.107. None of the interaction effects were significant, for Site and Site Order, *F*(2,22) = 0.669, *p* = 0.522, η^2^ = 0.057; for Site and Stress Level, *F*(2,22) = 1.544, *p* = 0.236, η^2^ = 0.123.

The main effect of Site on TINN was not significant, *F*(2,22) = 0.991, *p* = 0.387, η^2^ = 0.083. There was no significant main effect of either Site Order, *F*(1,11) = 0.551, *p* = 0.474, η^2^ = 0.048, or Stress Level, *F*(1,11) = 0.603, *p* = 0.454, η^2^ = 0.052. None of the interaction effects were significant, for Site and Site Order, *F*(2,22) = 0.158, *p* = 0.782, η^2^ = 0.014; for Site and Stress Level, *F*(2,22) = 0.459, *p* = 0.638, η^2^ = 0.040.

With respect to the frequency-domain, we did not find a significant main effect of Site on the Low Frequency signal, *F*(2,22) = 0.156, *p* = 0.857, η^2^ = 0.014, nor the High Frequency signal, *F*(2,22) = 2.614, *p* = 0.096, η^2^ = 0.192. Site Order seemed to have a marginally significant effect on the Low Frequency signal, *F*(1,11) = 4.206, *p* = 0.065, η^2^ = 0.277, but not on the High Frequency signal, *F*(1,11) = 0.216, *p* = 0.651, η^2^ = 0.019. No significant effects were observed of Stress Level on the Low Frequency or the High Frequency signal, *F*(1,11) = 0.529, p = 0.482, η^2^ = 0.046 and *F*(1,11) = 0.656, *p* = 0.435, η^2^ = 0.056, respectively. The interaction effect between Site and Site Order was not significant for the low Frequency signal, *F*(2,22) = 0.291, *p* = 0.750, η^2^ = 0.026, nor for the High Frequency signal, *F*(2,22) = 0.255, *p* = 0.777, η^2^ = 0.023. Finally, no significant interaction was observed between Site and Stress Level for the Low Frequency signal, *F*(2,22) = 0.385, *p* = 0.685, η^2^ = 0.034, nor for the High Frequency signal, *F*(2,22) = 2.610, *p* = 0.096, η^2^ = 0.192.

#### Electrodermal Activity

We found no significant effects on the phasic EDA activity (the number of significant skin conductance responses, nSCR), nor on the measure of tonic EDA activity.

The main effect of Site on nSCR was not significant, *F*(2,58) = 0.414, *p* = 0.663, η^2^ = 0.014. There was no significant main effect of either Site Order, *F*(1,29) = 0.638, *p* = 0.431, η^2^ = 0.022, or Stress Level, *F*(1,29) = 0.821, *p* = 0.372, η^2^ = 0.028. The interaction effects were not significant, for Site and Site Order, *F*(2,58) = 1.068, *p* = 0.350, η^2^ = 0.036; or for Site and Stress Level, *F*(2,58) = 0.657, *p* = 0.522, η^2^ = 0.022.

For the within-subjects effects on tonic EDA, we report Greenhouse–Geisser correct results, as Mauchley’s test of sphericity was significant [*W* = 0.382, 2(2) = 26.96, *p* < 0.001]. There was no significant main effect of Site, *F*(1.24,35.84) = 0.119, *p* = 0.785, η^2^ = 0.004. There was no significant main effect of either Site Order, *F*(1,29) = 0.325, *p* = 0.573, η^2^ = 0.011, or Stress Level, *F*(1,29) = 0.030, *p* = 0.865, η^2^ = 0.001. The interaction effects were not significant, for Site and Site Order, *F*(1.24,35.84) = 0.421, *p* = 0.563, η^2^ = 0.014; for Site and Stress Level, *F*(1.24,35.84) = 0.671, *p* = 0.449, η^2^ = 0.023.

#### EEG Response

**FIGURE 4:**
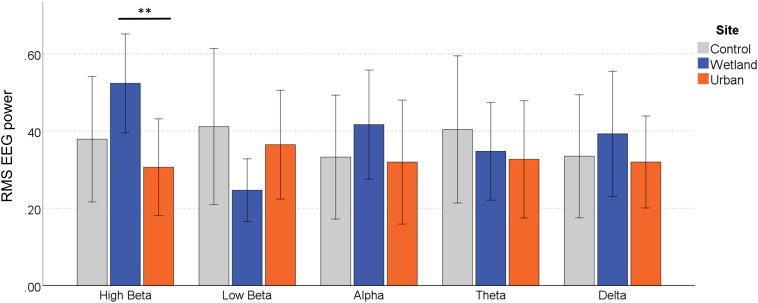
EEG response in the different frequency bands during exposure to the wetland, urban and control sites. Error bars indicate 95% confidence intervals. Statistical significance is indicated by ^*^p < 0.05 level, and ^∗∗^*p* < 0.01, corrected for multiple comparisons (Sidak).

For the effect of Frequency Band, we report Greenhouse–Geisser correct results, as Mauchley’s test of sphericity was significant [*W* = 0.145, 2(9) = 18.20, *p* = 0.035]. There was no significant main effect of Site on the RMS of the EEG signal, *F*(2,22) = 0.656, *p* = 0.529, η^2^ = 0.056, and there were no systematic differences between the Frequency Bands, *F*(2.28,25.02) = 0.418, *p* = 0.795, η^2^ = 0.037. Site Order also did not affect the EEG signal, *F*(1,11) = 0.614, *p* = 0.450, η^2^ = 0.053 and neither did Stress Level, *F*(1,11) = 1.667, *p* = 0.223, η^2^ = 0.132. However, of key interest is the interaction effect between Site and Frequency Band, which indicates differential environmental effects depending on the EEG band, *F*(8,88) = 2.195, *p* = 0.035, η^2^ = 0.166. Simple effects analysis showed that EEG responses varied by site only in the High Beta band, *F*(2,10) = 6.320, *p* = 0.017, η^2^ = 0.558. Significantly stronger signals were observed in the wetland versus the urban setting (*p* = 0.015), whereas they only marginally differed from the control setting (*p* = 0.076), and the urban and control setting also did not differ (*p* = 0.654) (Figure 4). None of the other frequency bands showed any differences in EEG signal [low-beta, *F*(2,10) = 2.482, *p* = 0.133, η^2^ = 0.332, alpha, *F*(2,10) = 0.691, *p* = 0.524, η^2^ = 0.121, Theta, *F*(2,10) = 0.264, *p* = 0.773, η^2^ = 0.050 and Delta, *F*(2,10) = 0.349, *p* = 0.714, η^2^ = 0.065).

None of the other two-way interactions were significant: Site by Site Order, *F*(2,22) = 2.729, *p* = 0.087, η^2^ = 0.199, Site by Stress Level, *F*(2,22) = 0.30, *p* = 0.971, η^2^ = 0.00, Frequency Band by Order, *F*(2.28,25.02) = 0.253, *p* = 0.805, η^2^ = 0.023, and Frequency Band by Stress Level, *F*(2.28,25.02) = 1.309, *p* = 0.291, η^2^ = 0.106. The three way interaction between Site, Frequency Band and Order was not significant, *F*(8,88) = 1.652, *p* = 0.122, η^2^ = 0.131, and neither was the interaction between Site, Frequency Band and Stress Level, *F*(8,88) = 0.533, *p* = 0.829, η^2^ = 0.046.

#### Self-Reported Mood States

For the positive subscale of the PANAS, we found a significant main effect of Site, *F*(2,58) = 3.698, *p* = 0.031, η^2^ = 0.113, demonstrating the strongest positive feelings were reported in the wetland setting (*M* = 32.86, *SD* = 11.55), followed by the control (*M* = 30.29, *SD* = 10.03), and then the urban (*M* = 28.18, *SD* = 10.92). There was no significant main effect of either Site Order, *F*(1,29) = 0.186, *p* = 0.669, η^2^ = 0.006, or Stress Level, *F*(1,29) = 2.434, *p* = 0.130, η^2^ = 0.077. None of the interaction effects were significant, for Site and Site Order, *F*(2,58) = 1.588, *p* = 0.213, η^2^ = 0.052; for Site and Stress Level, *F*(2,58) = 0.083, *p* = 0.921, η^2^ = 0.003.

**FIGURE 5:**
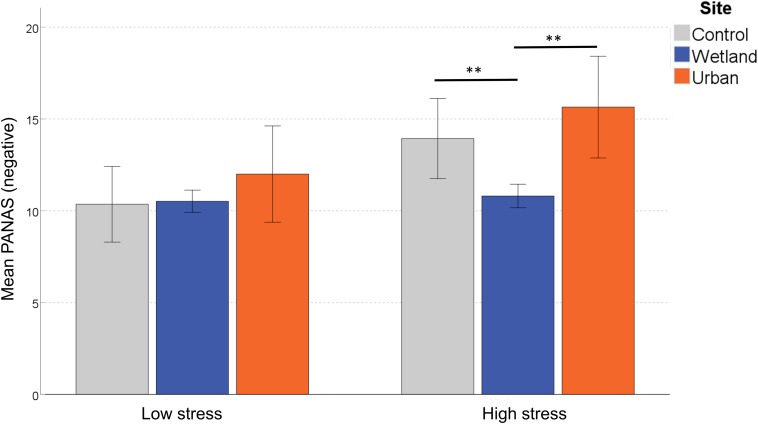
Differences in self-reported negative mood following exposure to the three sites. Error bars indicate 95% confidence intervals. Statistical significance is indicated by ^*^p < 0.05 level, and ^∗∗^*p* < 0.01, corrected for multiple comparisons (Sidak).

For the negative subscale of the PANAS, we found a significant main effect of Site, *F*(2,58) = 8.671, *p* = 0.001, η^2^ = 0.230. There was no significant main effect of Site Order, *F*(1,29) = 0.209, *p* = 0.651, η^2^ = 0.007, but the main effect of Stress Level was significant, *F*(1,29) = 5.352, *p* = 0.028, η^2^ = 0.156. Because we also found an interaction effect between Site and Stress Level, *F*(2,58) = 0.083, *p* = 0.921, η^2^ = 0.003, we conducted a simple effects analysis, which demonstrated that for the subgroup classified as ‘self-reported low stress,’ PANAS negative scores did not differ between sites, *F*(2,28) = 1.188, *p* = 0.320, η^2^ = 0.078, but those classified as more stressed reported significant differences, *F*(2,28) = 8.585, *p* = 0.001, η^2^ = 0.380. Pairwise comparisons demonstrated significantly fewer negative feelings in the wetland setting compared with both the control (*p* = 0.006) and the urban setting (*p* = 0.002; both corrected for multiple comparisons) (Figure 5).

## Discussion

Mental health is negatively associated with urban living (Pedersen and Mortensen, 2001; Van Os et al., 2004; Peen et al., 2010; Lederbogen et al., 2011) and positively associated with residential proximity to green and blue urban space (White et al., 2013; Alcock et al., 2014; Gascon et al., 2015). There is a growing interest across a broad set of disciplines – from public health to conservation biology – into how natural features might mitigate the impact of stress induced by urban environments (Thompson et al., 2014). Advances in wearable technologies now allows those managing blue and green spaces to monitor people’s responses in real time and *in situ*. Here, we examine acute physiological and psychological stress responses during short-term exposure to a typical urban setting and an urban wetland in London, providing insight into the feasibility of using low-cost wearable technology the monitor the HWB co-benefits of urban blue-green space.

Of the various physiological measures we acquired, only the HR response was found to be significantly different between exposure conditions. While acute cardiovascular responses to nature have been noted in earlier studies (e.g., Lanki et al., 2017), novel in our study was the finding that this effect was contingent upon participants’ pre-exposure stress levels. Participants with higher than average levels of stress in the week prior to the experiment experienced an increase in HR in the urban, but not the wetland setting, relative to the baseline acquired indoors. As increased HR is an indicator of general physiological arousal, exposure to urban environments appears to cause a stress response, while wetland setting has a mitigating effect. In contrast to previous studies in which decreased HR was observed during exposure to green spaces relative to a control (e.g., Song et al., 2013; South et al., 2015; Grazuleviciene et al., 2015; Lanki et al., 2017), our findings suggest that acute exposure to blue–green spaces may induce a subtle and short-term moderation of cardiovascular stress reactivity. With the current experimental design we can only correlate this finding with the blue–green environment exposure, limiting the alignment of our observations with existing theories in the field e.g., the physiological and emotional stress moderation posited by Stress Recovery Theory (Ulrich, 1983). Further research is required in order to identify the mechanisms linking specific facets of the wetland environments to this observed moderation. Only with such advances can we begin to understand how the negative impacts of stressors associated with urban life may be mitigated through the expansion of BGI.

In addition to the physiological metrics, we also asked participants to report changes in mood states using a validated self-report instrument. Again, effects on mood state varied depending on participants’ pre-exposure stress levels. Those with self-reported elevated stress levels in the week prior also reported a reduction in negative feelings after 10 min in the wetland compared with both the urban and control settings. This suggest at least a temporary improvement in subjectively experienced mood for those experiencing high stress in everyday life. In a similar vein, Roe and Aspinall (2011b) found more beneficial outcomes for adults in poor mental health during a rural walk. Korpela et al. (2008) and Ottosson and Grahn (2008) suggested that nature may have stronger restorative powers for people experiencing greater emotional stress. The evidence thus suggests that exposure to blue-green spaces can improve negative mood states. Interestingly, irrespective of whether participants were stressed prior to the experiment, a universal enhancement of positive mood states was observed following exposure to the wetland site, with people reporting feeling ‘attentive,’ ‘inspired,’ ‘strong’ and ‘enthusiastic.’ This finding confirms the positive association between blue-green space provision and hedonic wellbeing or life satisfaction (for a review, see: Houlden et al., 2017). Our findings therefore suggest that, in terms of subjective experience, exposure to urban blue space may have a dual effect of reducing stress and promoting more positive mood states, at least temporarily.

Based on the evidence that the wetland setting may induce both psychological and physical restoration, at least in those susceptible to stress, we had also expected to find a site-dependent moderation of brain activity. In particular, a state of relaxation or mediation would be associated with an increase in power across the alpha and theta frequency bands. However, only the high beta frequency band increased during wetland exposure, consistent with a state of enhanced attention. Under more controlled conditions (where participants passively viewed a series of videos), Olszewska-Guizzo et al. (2018) found increased power in the beta frequency band, located in temporal brain during the observation of ‘contemplative’ spaces relative to ‘non-contemplative’ outdoor spaces. They interpreted this pattern of brain activity as indicating more holistic perceptual processing and/or attention driven toward salient stimuli, associated with fascination. Increased beta-power could therefore be consistent with a restoration effect driven by involuntary attention shifts. There are good reasons, however, to be cautious when interpreting the EEG results. Few studies have attempted EEG measures *in situ* and across different environmental gradients, and results have been inconsistent. Previous studies using the Emotiv^®^ EEG technology applied proprietary algorithms that automatically process and translate the EEG signals into mental state indicators. Aspinall et al. (2015) found lower ‘frustration,’ ‘engagement’ and ‘arousal,’ and higher ‘meditation’ when young participants walked into green space, and higher ‘engagement’ when moving out of it. Neale et al. (2017) reported that urban green space enhanced older participants’ ‘engagement,’ whereas typical urban spaces promoted EEG activity associated with ‘excitement.’ There is – to our knowledge – no published research that reliably links these ‘mental state’ indicators with specific neural activity. The unconfirmed validity of this EEG analysis method led us instead to process the raw signals to derive the frequency band data within the EEG signal. Despite our attempts to improve electrode conductance (e.g., using glycerol/saline solution as recommended for longer measurement periods), our EEG observations are consistent with significant signal deterioration. The outdoor setting and an individual’s movements whilst walking increased the likelihood of electrode displacement and signal interference. These effects cannot be resolved through *post hoc* statistical artifact correction. Significant loss of signal quality restricted the quantity of data available for processing. In addition, the low spatial resolution of the 14-channel Emotiv^®^ system precludes analysis of signal origins, further complicating the interpretation of the data (but see Chen et al., 2015 for an example of a basic connectivity analysis demonstrating increased synchronicity in EEG waves during exposure to a natural environment). We report on averaged values across the full electrode network, and this broad-stroke approach, further compromised by the noisy signal due to acquisition problems, may have failed to capture the more subtle variations in brain function. Perhaps unsurprisingly, the user-friendliness and intuitive appeal of easy-to-use, low-cost mobile EEG systems is traded off against data quality. Research-grade systems are undoubtedly superior, but they require substantial training and may not be suitable for most mobile applications and limited budgets, and with some modifications acceptable accuracy can be achieved with low-cost systems (e.g., see Mavros et al., 2016). More research is required to establish validated protocols, optimize the electrode setup, and/or develop a range of bespoke modifications to increase signal quality and overall stability for mobile experiments.

We experienced similar technical difficulties with the acquisition and processing of some of the other psychophysiological metrics, which may have produced the negative findings on the HRV measures and indicators of EDA. Despite promising results in laboratory settings using images and 3-D simulations (e.g., Gladwell et al., 2012; Annerstedt et al., 2013; Brown et al., 2013), studies that have examined the effect of green space exposure on HRV (no studies have specifically examined blue spaces) report mixed findings (Kondo et al., 2018b). We hypothesize that this is due, at least in part, to the heterogeneity in how HRV is quantified. When derived using PPG, rather than ECG, measurements, HRV analysis involves several sequential calculations and each may apply different techniques (Michael et al., 2017). Despite the practical advantages, PPG devices may not (yet) provide useable HRV data suitable for monitoring and evaluating HWB benefits. We concur with Gidlow et al. (2016) who suggest that the level of noise in HRV recordings obtained under field conditions hampers our ability to discern meaningful and/or statistically significant differences in HRV response to changes in environmental exposure.

Contrary to our expectations, we also found no difference in EDA between the exposure conditions, despite the fact that skin conductance is less susceptible to the confounding effect of concurrent physical activity, and more closely linked to psychological arousal or stress. Evidence from other studies suggests that low-cost mobile sensors can provide data on par with research-grade systems (Torniainen et al., 2015; Zangróniz et al., 2017). One exploratory study found correlations between EDA activity and urban design features, demonstrating in particular that traversing green spaces was associated with reduced stress responses (Fathullah and Willis, 2018). However, as with the HRV data, a likely explanation for the negative finding is the low EDA signal quality. Environmental factors such as temperature and humidity, and a participant’s sweat production, are known to affect EDA, which can lead to inconsistent results, especially in field experiments and when analyzing short epochs (e.g., Doberenz et al., 2011). Our analysis and interpretation of the EDA data may also have been hampered by an inadequate period for the baseline measure. The 10 min baseline obtained in the control room did not provide sufficient insight into the tonic aspect of the signal, which varies between individuals and across normal diurnal patterns (e.g., Hot et al., 1999). In future studies, an observational approach in which participants wear the Empatica E4 monitor for a pre-experiment period of 24 h (or longer) combined with GPS location services could provide rich data that would allow direct comparisons in responses to various urban design features in an ecologically valid manner.

In addition to the technological challenges outlined earlier, we recognize a number of limitations associated with our experimental design. Time constraints limited the environmental exposure to a single wetland site, comprising both blue and green elements, and an urban site. The environments vary on a great number of variables, across a range of perceptual domains. This precludes judgments on the specificity of the observed effects. It remains unclear whether exposure to other types of blue and/or green spaces could deliver similar benefits. The absence of a green environment control (e.g., a municipal parkland) means we are unable to determine the impact of urban wetland habitat as opposed to any other non-built environment. As such our conclusions are drawn using more generic terms around ‘blue–green space’ as opposed to ‘blue space.’ Future studies should incorporate a greater range of environmental stimuli to identify specific elements producing strong effects, what the role of biodiversity is (Dean et al., 2011; Dallimer et al., 2012; Clark et al., 2014) and whether perceived or ‘objective’ quality and type of habitat matters (Wheeler et al., 2015).

Due to the age range and anticipated variation in physical condition within the participant sample, any psychophysiological measure would be subject to large inter-individual variation, which introduces measurement noise and impacts the detection of statistically significant differences in a relatively small sample. While our sample size compares favorably with similar quasi-experimental studies (Kondo et al., 2018b), we caution against extrapolation, and emphasize the need to re-examine these effects at different sites and with larger, and possibly more homogeneous samples. Inter-individual variation may be mitigated by obtaining more robust baselines against which exposure responses can be compared, e.g., through unobtrusive 24-h measurements (Kondo et al., 2018b). Such recordings are certainly possible with the PPG wristband, but for the EEG measure, this remains impractical for a wide range of applications as the individual needs to carry the acquisition device (e.g., laptop) in addition to wearing the headset. Furthermore, 70% percent of participants we queried (21/30) reported discomfort from prolonged wearing of the headset, with initial signs of irritation or physical discomfort occurring during the experimental session. This may have caused both technical problems (e.g., more movement artifacts) and influenced the mental state of participants, confounding the EEG results. Our experimental setup may have therefore tested the limits of the current technology.

Our categorization of participants into self-reported stressed and non-stressed (defined against normative UK data; Henry and Crawford, 2005) has limitations because it does not fully encompass the complexity of the environment-stress dynamic at an individual level. Differing environments, personal experience and individual capabilities are likely to interact to produce differential psychological responses. For example, we have assumed that the ‘self-reported high stress’ subset did so based on a depletion in personal resource and that the ‘self-reported low stress’ subset did so through a perceived lack of depletion. This fails to recognize the role of ‘instoration’ (the accruing of adaptive personal resources (e.g., self-esteem) beyond the restoration of a deficit (Hartig et al., 1996). We also do not know the role the environment plays in instoration. For example those reporting as ‘low stress’ might have experienced improved mental health by residing in areas with good green or green-blue space provision (White et al., 2013; Alcock et al., 2014). As such there is a need for more instorative studies on the role the environment plays in building health resilience (Roe, 2008; Roe and Aspinall, 2011a). Such studies would help tackle important evidence gaps on the mechanisms around how differing natural and built environments (or facets of those environments) contribute to building or depleting individual resources. For example what dose or exposure (Barton and Pretty, 2010; Cox et al., 2017; White et al., 2019) or level of biodiversity (Aerts et al., 2018) might be required to build resilience, and for whom? Such studies might also advance findings on the beneficial effects of nature that manifest even when participants are not exposed to any psychological depletion (Beute and De Kort, 2014). Such advances have important policy and advocacy implications for those agitating for greater BGI provision in urban environments for health and broader ecosystem services that benefit people.

As highlighted by Roe and Aspinall (2011b), the quasi-experimental design employed in field experiments presents a number of limitations. First, it is difficult to control for the many confounding variables that impact on participant comfort and experience in outdoor environments. We managed this to some extent with the timing of the experiment. We had favorable weather and a capacity to stipulate testing days without rain, wind or cold temperatures. Cloud cover was the most variable weather feature, and we do anticipate that differences in the level of sunshine are likely to stimulate variations in both physiological and psychological response, as well as producing additional risk of sweat-related artifacts on the EEG and EDA measures. Variation between morning, lunch and afternoon sessions potentially introduced diurnal physiological differences. In the urban environment, when the general public unexpectedly interrupted testing, we retested participants, however, we were unable to control the number of passers-by in both urban and wetland and how this may have affected participants. Another possible confounding variable is prior experience and knowledge of the site and surrounds.

Our experimental manipulation mimics the incidental nature of exposure to BGI in urbanites (Keniger et al., 2013; Beery et al., 2017). The results mainly illustrate the psychological (mood-based) effects of one-off blue-green space exposures in urban settings, and highlight potential concomitant physiological effects that require more detailed investigation. An interesting further research direction concerns the issue of whether and how the benefits of incidental exposure accumulate over time and across a life course. It also remains to be demonstrated whether there are any particular environmental characteristics or design features in managed settings that produced the enhanced psychological benefits. This level of detail was beyond the scope of the current study, but a deeper understanding will be required to adequately inform and optimize the implementation of BGI for mental and physical health salutogenesis in urban areas.

## Conclusion

In this experiment, we aimed to test the salutogenic potential of an urban wetland, using commercially available, low cost wearable technologies. Whilst only the most robust physiological measure showed a differential response to the wetland environment compared to the urban environment, our results are consistent with the hypothesis that exposure to blue–green space promotes stress recovery. People that are already experiencing a high level of stress may be at greater risk of developing health problems due to an increased physiological stress response in busy urban environments. At the same time, individuals experiencing high stress appear to derive some relief from negative mood states during green–blue space exposure, which also enhances positive feelings in most people. Further research is required to examine the degree to which the provision of BGI in urban environments has preventative and curative benefits, and to validate whether incidental exposure is sufficient to achieve meaningful effects over long-term timeframes and across a larger number of response variables.

Finally, our experience indicates that wearable monitoring devices have an intuitive appeal with various stakeholders, including the academic community, conservation organizations, the public, and results may be persuasive with policy makers. We urge vigilance amongst those considering implementing this technology, to recognize the risk it poses in becoming a fad across the environment sector, as has occurred with other potential solutions in the past (Redford et al., 2013). The drive toward an evidence-based approached risks being hijacked by such a fad if the challenges of successfully navigating research-implementation ‘spaces’ (Toomey et al., 2017) are subsumed by the latest technological wizardry. We must therefore remain vigilant as we work toward expanding the evidence base on public health benefits as a robust argument for the conservation of blue and green space in our urban environment.

## Data Availability

The dataset generated for this study can be found on a dedicated page within the Open Science Framework domain, maintained by the Center for Open Science (COS) (https://osf.io/kfxsr/).

## Ethics Statement

This research was conducted in accordance with the RCUK Policy and Guidelines on Governance of Good Research Conduct (updated in 2017) and the guidelines set out by Imperial College London (https://www.imperial.ac.uk/research-and-innovation/about-imperialresearch/research-integrity/ethics/human/). All data was stored in accordance with the UK Data Protection Act of 1988 (which was in force when the project commenced) and the EU General Data Protection Regulation which came into force in 2018. An internal review process within the Department of Life Sciences was used to investigate the ethical dimensions of the proposed research. Further elevation to a full review by the Imperial College Research Ethics Committee (ICREC) was not required on the basis of this initial assessment. The above process followed standard procedures for the assessment of low-risk, non-medical human research studies, as approved by the Head of Department of Life Sciences and the Head of Regulatory Compliance (within the Joint Research Compliance Office) at Imperial College London.

## Author Contributions

JR, AV, RC, and AK conceived the research and secured funding for the project. JR and AV developed the study design, oversaw the data collection and analysis, and drafted the initial version of the manuscript. JR, AV, and ES analyzed and interpreted the data. VH contributed to participant recruitment and data collection. CN contributed to the development of the study design and data analysis. All authors contributed to the writing and editing of the manuscript.

## Conflict of Interest Statement

The authors declare that the research was conducted in the absence of any commercial or financial relationships that could be construed as a potential conflict of interest.

## Footnotes

1 wwt.org.uk

2 www.metoffice.gov.uk
