# Supplementary material for: The Application of Wearable Technology to Quantify Health and Wellbeing Co-benefits From Urban Wetlands
Source: Front Psychol. 2019 Aug 13;10:1840. doi: 10.3389/fpsyg.2019.01840 (PMC6700336; doi:10.3389/fpsyg.2019.01840)

Supplementary Material

**Supplementary table 1.** The number of HRV observations (i.e. epochs) classified by condition and by data quality. To retain an observation for further analysis, we required at least 5 minutes of good data after artefact rejection.

|  | **Quality** |  |  |  |
| --- | --- | --- | --- | --- |
| **Site** | **Good/sufficient data** | **Poor/insufficient data** | **Missing/data not recorded** | **Total N** |
| **Control** | 29 | 5 | - | 34 |
| **Urban** | 22 | 12 | - | 34 |
| **Wetland** | 18 | 15 | 1 | 34 |
| **Total epochs** | 69 | 32 | 1 |  |

**Supplementary Table 2.** Independent samples t-test for gender differences on the baseline measures of stress and nature relatedness. The only significant differences between men and women were observed on Nature Relatedness, as depicted in Supplementary Figure 2.

|  | Levene’s Test of Equality of Variances | T-test for equality of means | | | | | | |
| --- | --- | --- | --- | --- | --- | --- | --- | --- |
| Measure |  | t | df | p | Mean difference | SD | 95% Confidence Interval of the Difference | |
| Holmes-Rahe Social Readjustment Scale | Equal variances assumed | -1.54 | 31 | 0.134 | -109.74 | 71.23 | -255.02 | 35.53 |
| DASS-21 score (depression subscale) | Equal variances assumed | -1.58 | 31 | 0.123 | -1.74 | 1.10 | -3.98 | 0.50 |
| DASS-21 score (anxiety subscale) | Equal variances assumed | -0.57 | 31 | 0.574 | -0.37 | 0.64 | -1.68 | 0.95 |
| DASS-21 score (stress subscale) | Equal variances assumed | -0.33 | 31 | 0.743 | -0.52 | 1.56 | -3.69 | 2.66 |
| Nature relatedness score (NR-Self) | Equal variances assumed | 3.25 | 32 | 0.003 | 4.79 | 1.47 | 1.78 | 7.79 |
| Nature relatedness score (NR-Perspective) | Equal variances not assumed | 3.39 | 18.31 | 0.003 | 4.48 | 1.32 | 1.71 | 7.25 |
| Nature relatedness score (NR-Experience) | Equal variances assumed | 0.63 | 32 | 0.536 | 0.97 | 1.55 | -2.18 | 4.12 |

**Supplementary Table 3.** Correlations between baseline variables. * Correlation is significant at the p < 0.05 level (2-tailed); ** correlation is significant at the p< 0.01 level (2-tailed).

|  | | a | b | c | d | e | f | g | h |
| --- | --- | --- | --- | --- | --- | --- | --- | --- | --- |
| Age of participant ^a^ | Pearson Correlation | 1 | -0.257 | -0.160 | -0.257 | -0.054 | .426^*^ | 0.280 | 0.212 |
|  | Sig. (2-tailed) |  | 0.171 | 0.399 | 0.170 | 0.776 | 0.017 | 0.127 | 0.253 |
|  | N | 31 | 30 | 30 | 30 | 30 | 31 | 31 | 31 |
| Holmes-Rahe Social Readjustment Scale ^b^ | Pearson Correlation | -0.257 | 1 | 0.199 | 0.265 | .472^**^ | -0.220 | -0.264 | -0.179 |
|  | Sig. (2-tailed) | 0.171 |  | 0.275 | 0.142 | 0.006 | 0.218 | 0.137 | 0.319 |
|  | N | 30 | 33 | 32 | 32 | 32 | 33 | 33 | 33 |
| DASS-21 score (depression subscale) ^c^ | Pearson Correlation | -0.160 | 0.199 | 1 | .754^**^ | .700^**^ | -0.033 | 0.139 | 0.116 |
|  | Sig. (2-tailed) | 0.399 | 0.275 |  | 0.000 | 0.000 | 0.856 | 0.442 | 0.520 |
|  | N | 30 | 32 | 33 | 33 | 33 | 33 | 33 | 33 |
| DASS-21 score  (anxiety subscale) ^d^ | Pearson Correlation | -0.257 | 0.265 | .754^**^ | 1 | .680^**^ | -0.060 | 0.095 | -0.126 |
|  | Sig. (2-tailed) | 0.170 | 0.142 | 0.000 |  | 0.000 | 0.741 | 0.598 | 0.486 |
|  | N | 30 | 32 | 33 | 33 | 33 | 33 | 33 | 33 |
| DASS-21 score  (stress subscale) ^e^ | Pearson Correlation | -0.054 | .472^**^ | .700^**^ | .680^**^ | 1 | -0.033 | 0.107 | -0.141 |
|  | Sig. (2-tailed) | 0.776 | 0.006 | 0.000 | 0.000 |  | 0.855 | 0.555 | 0.434 |
|  | N | 30 | 32 | 33 | 33 | 33 | 33 | 33 | 33 |
| Nature relatedness score – Self ^f^ | Pearson Correlation | .426^*^ | -0.220 | -0.033 | -0.060 | -0.033 | 1 | .629^**^ | .667^**^ |
|  | Sig. (2-tailed) | 0.017 | 0.218 | 0.856 | 0.741 | 0.855 |  | 0.000 | 0.000 |
|  | N | 31 | 33 | 33 | 33 | 33 | 34 | 34 | 34 |
| Nature relatedness score – Perspective ^g^ | Pearson Correlation | 0.280 | -0.264 | 0.139 | 0.095 | 0.107 | .629^**^ | 1 | 0.256 |
|  | Sig. (2-tailed) | 0.127 | 0.137 | 0.442 | 0.598 | 0.555 | 0.000 |  | 0.145 |
|  | N | 31 | 33 | 33 | 33 | 33 | 34 | 34 | 34 |
| Nature relatedness score – Experience ^h^ | Pearson Correlation | 0.212 | -0.179 | 0.116 | -0.126 | -0.141 | .667^**^ | 0.256 | 1 |
|  | Sig. (2-tailed) | 0.253 | 0.319 | 0.520 | 0.486 | 0.434 | 0.000 | 0.145 |  |
|  | N | 31 | 33 | 33 | 33 | 33 | 34 | 34 | 34 |

**Supplementary Figure 1.** Outlier analysis for the EEG data. Numbers indicate participant IDs. We removed 30 data points prior to statistical analysis of the Site effects on the EEG.


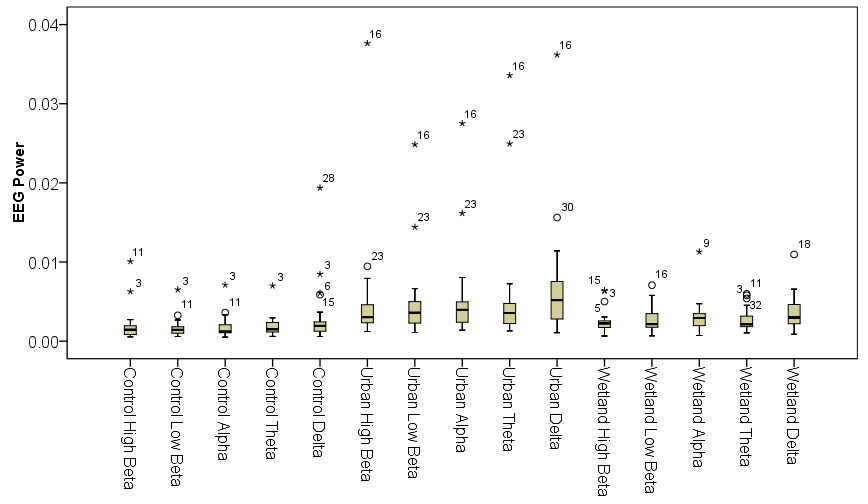


**Supplementary Figure 2.** Gender differences in Nature Relatedness. ** Indicates p < .01.


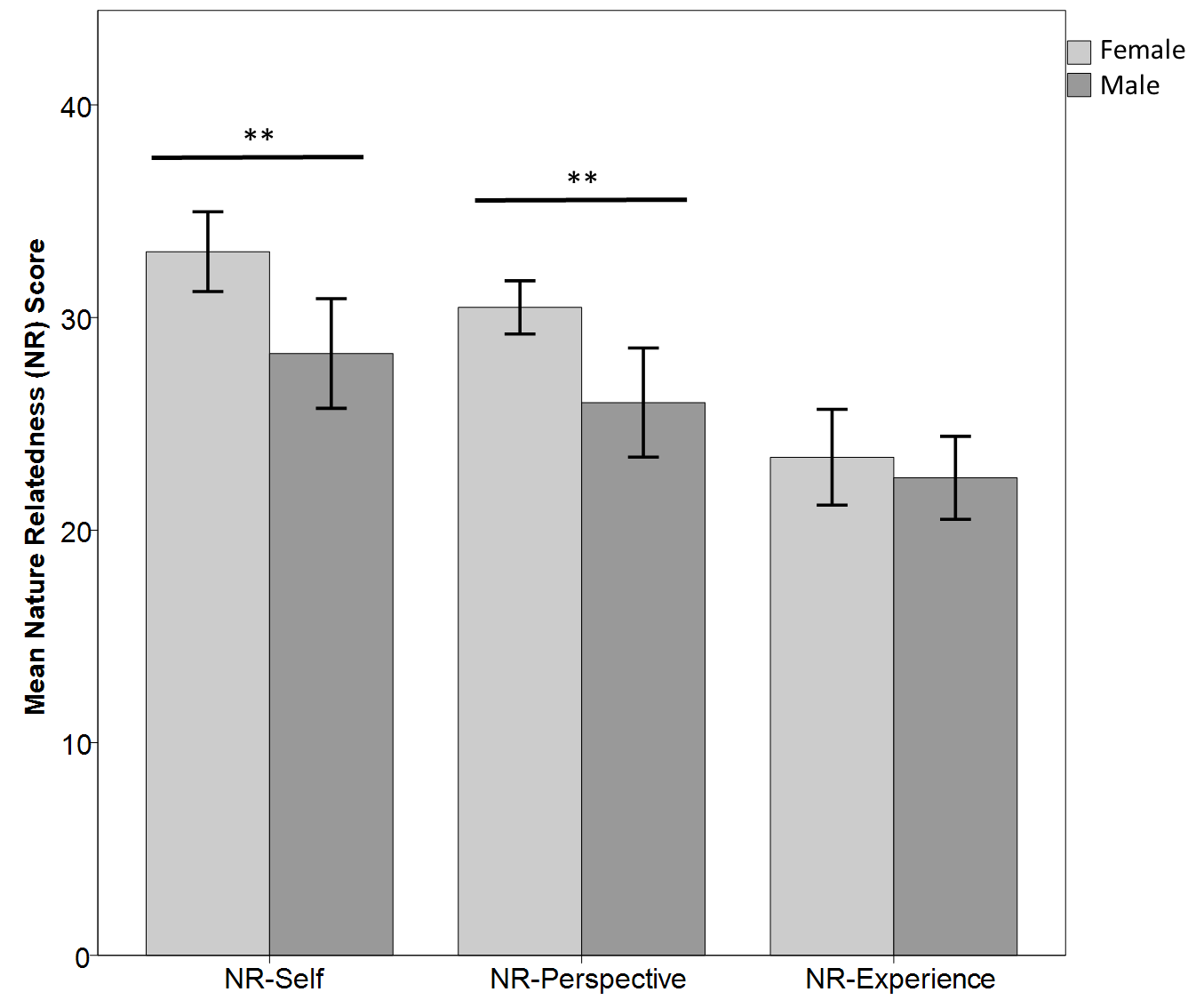

Supplement: Supplementary file 1 [file Table_1.DOCX]
